# Delay in the diagnosis and treatment of pulmonary tuberculosis in Uzbekistan: a cross-sectional study

**DOI:** 10.1186/s12879-014-0624-y

**Published:** 2014-11-25

**Authors:** Tatiana V Belkina, Doniyor S Khojiev, Mirzagaleb N Tillyashaykhov, Zinaida N Tigay, Marat U Kudenov, Jurjen Duintjer Tebbens, Jiri Vlcek

**Affiliations:** Department of Social and Clinical Pharmacy, Faculty of Pharmacy in Hradec Kralove, Charles University in Prague, 1203 Heyrovskeho, Hradec Kralove, 50005 Czech Republic; Ministry of Health of the Republic of Karakalpakstan, 152 Doslyk guzari, Autonomous Republic of Karakalpakstan, Nukus, 142005 Uzbekistan; Republican Specialized Scientific and Practical Medical Centre for Phthisiology and Pulmonology, 1 Alimova, Tashkent, 100086 Uzbekistan; Department of Biophysics and Physical Chemistry, Faculty of Pharmacy in Hradec Kralove, Charles University in Prague, 1203 Heyrovskeho, Hradec Kralove, 50005 Czech Republic; Institute of Computer Science Academy of Sciences of the Czech Republic, 271 Pod Vodarenskou vezi, Prague 8, 18207 Czech Republic

## Abstract

**Background:**

Early diagnosis and prompt effective therapy are crucial for the prevention of tuberculosis (TB) transmission, particularly in regions with high levels of multi-drug resistant TB. This study aimed to evaluate the extent of delay in diagnosis and treatment of TB in Uzbekistan and identify associated risk factors.

**Methods:**

A cross-sectional study was performed on hospital patients with newly diagnosed TB. The time between the onset of respiratory symptoms and initiation of anti-TB treatment was assessed and delays were divided into patient, health system and total delays. Univariable and multivariable logistic regression analysis was used to evaluate determinants of diagnostic and treatment delay.

**Results:**

Among 538 patients enrolled, the median delay from onset of symptoms until treatment with anti-TB drugs was 50 days. Analysis of the factors affecting health-seeking behaviour and timely treatment showed the presence of the patient factor. Self-medication was the first health-seeking action for 231 (43%) patients and proved to be a significant predictor of delay (p = 0.005), as well as coughing (p = 0.009), loss of weight (p = 0.001), and visiting private and primary healthcare facilities (p = 0.03 and p = 0.02, respectively).

**Conclusion:**

TB diagnostic and treatment delay was mainly contributed to by patient delay and should be reduced through increasing public awareness of TB symptoms and improving public health-seeking behaviour for timely initiation of anti-TB treatment. Efforts should be made to minimise irrational use of antibiotics and support interventions to restrict over-the-counter availability of antibiotics.

## Background

Tuberculosis (TB) is one of the oldest human diseases, yet it still cannot be defeated and remains second only to HIV as a leading cause of death from infectious diseases worldwide. In 2012, there were an estimated 8.6 million new TB cases and 1.3 million deaths [1]. The TB epidemic has been fuelled by a surge in HIV-TB co-infection and compounded by the growing emergence of multi- and extensively drug resistant (M/XDR) TB strains, with a high prevalence in the former Soviet Union countries, especially in Central Asia [2],[3]. Uzbekistan is one of the four Central Asian states among the 27 identified by the World Health Organization (WHO) with the highest burden of multi-drug resistant TB (MDR-TB).

The highest MDR-TB incidence rate exists in North-western Karakalpakstan, where the immunity of the population has been undermined by the Aral Sea ecological catastrophe. Also cotton growing regions suffer particularly because the immunity of the local people has been adversely affected by unlimited application of pesticides [4].

Substantial gains have been made in reducing TB morbidity and mortality in the past decade. New directives have been approved, TB control programmes have been implemented to prevent transmission in the community, and a good-quality directly observed therapy short-course strategy (DOTS) has been expanded throughout the country with 100% regional coverage [5].

However, the current positive trend is insufficient to ensure stable and efficient control of TB. The TB surveillance and monitoring report in Europe and Central Asia yielded an estimated 14,787 incident cases of TB in Uzbekistan in 2012 [3]. There were an estimated 2,400 (23%) new and 1,600 (62%) retreatment MDR-TB cases among notified TB patients [6]. In 2012, 1,728 MDR-TB cases were detected among patients with reported TB [6]. This is 43.2% of all estimated cases, indicating a low detection rate of MDR-TB. Given the substantial incidence of MDR-TB in Uzbekistan, delay in diagnosis increases the risk of onward transmission through untreated patients leading to aggravation of the situation in the region.

Delay in TB diagnosis and treatment has been studied internationally and several definitions of delay have been used, but frequently applied definitions are delay due to the patient or healthcare system. To the best of our knowledge, the possible delays in diagnosis and initiation of anti-TB treatment have not been extensively researched in Central Asia, and there are limited data from the high MDR-TB-endemic countries of the former Soviet Union.

This article reviews the extent of delay in diagnosis and treatment of TB and associated risk factors that might be tackled to promote early diagnosis and improve TB prevention.

## Methods

### Setting and study design

Between August 2013 and January 2014, we conducted a cross-sectional study among newly diagnosed pulmonary TB patients in two cities of Uzbekistan, Tashkent and Nukus. In Tashkent, the study was conducted at the Republican Specialized Scientific and Practical Medical Centre for Phthisiology and Pulmonology providing treatment to patients from across the country. In Nukus, the main city of the Autonomous Republic of Karakalpakstan, two hospitals and one dispensary were chosen as study settings.

### Study population

We reviewed the TB Electronic Surveillance Case-based Management System (ESCM) database of all cases of culture-confirmed pulmonary TB registered in the selected facilities from August 2013 to January 2014. Paper-based inpatient and outpatient medical cards from adult patients (aged ≥15 years) were reviewed and all patients with newly diagnosed pulmonary TB were included in the study. We excluded those with recurrent TB.

The patients were interviewed using an adapted and slightly modified version of the WHO questionnaire developed for the assessment of TB diagnostic and treatment delay [7]. The questionnaire was pre-tested and administered by trained doctors and health workers. All interviews were performed in the Uzbek, Karakalpak or Russian language.

Verbally informed consent was obtained from patients prior to inclusion in the survey, and the study was approved by the Ethical Committee of Charles University in Prague, the Ministry of Health of the Republic of Karakalpakstan, and the Ethical Committee of the Republican Specialized Scientific and Practical Medical Centre for Phthisiology and Pulmonology.

### Definitions

Patients were considered to have pulmonary TB on the basis of clinical, pathological and radiological findings confirmed bacteriologically and histologically. The time between onset of respiratory symptoms and initiation of anti-TB treatment was assessed and delays were divided into three types. Patient delay was measured in days from the first onset of any TB symptom (e.g., cough, fever, weight loss, or night sweats) until the first presentation to the healthcare system (not necessarily a TB facility). Patient delays were allowed to be negative, indicating the situation in which regular healthcare visits detected TB infection before onset of symptoms. Healthcare delay was measured as the number of days from first presentation to the healthcare system until the start of TB treatment. Total delay was calculated as patient delay plus healthcare delay (and thus may also take negative values). Comorbidity was defined as underlying cardiovascular, gastrointestinal, pulmonary, immunologic or malignant disease. Self-medication was defined as use of any medication not prescribed by a healthcare professional. Antibiotics and other medications are available at pharmacies in Uzbekistan over-the-counter and without a prescription.

### Laboratory techniques

Analysis of sputum samples for smear microscopy, culture, drug-susceptibility testing (DST) and real-time PCR (Xpert MTB system) were conducted as per international guidelines [8]-[10] at the National Reference laboratory in Tashkent and at the Mycobacterial Laboratory in Nukus. Preliminary confirmation of acid-fast bacilli (AFBs) was performed using Ziehl-Neelsen staining while culturing was done using Lowenstein-Jensen medium. AFB strains were classified according to the WHO/International Union against Tuberculosis and Lung Disease scale: 1+ (10-99 AFBs per 100 fields), 2+ (1-10 AFBs per individual field), 3+ (10-100 AFBs per individual field), and 4+ (>100 AFBs per individual field) [11]. The BACTEC MGIT 960 system, based on the critical concentration method, was used for determining susceptibility to first-line drugs. DST for second line drugs was performed using the agar proportion method in Lowenstein-Jensen medium. Provider-initiated HIV testing is routinely recommended for all patients presenting with symptoms and signs of TB and TB diagnosed patients [12],[13]. MDR-TB and HIV-associated TB sputum samples were analysed using real-time PCR Xpert MTB assay.

### Data collection and analysis

The questionnaire included sociodemographic characteristics, risk factors of TB, comorbidity, and TB knowledge and attitudes. Follow-up data included history of TB treatment, such as a detailed description of diagnostic investigation process, first symptoms perceived by the patient, and health seeking actions. The patients were also asked to complete a number of questions measuring psychosocial aspects, for example, feeling ashamed about having TB, fear of social isolation and stigma. In addition, patient medical cards were reviewed for TB diagnostic information, such as date of diagnosis, date of treatment initiation, and laboratory results.

Univariable and multivariable logistic regression analysis was used to evaluate risk factors for patient, healthcare and total delays, which were dichotomised into delay versus non-delay. Following studies in comparable countries (e.g. Rabin et al. [14]), median delays were used as a cut-off. Variables included in the multivariable model were chosen according to the strategy of purposeful selection [15] based on behavioural and biological plausibility as well as statistical (p ≤ 0.2) criteria. The regression outcome was given by the estimated (adjusted) odds ratios and the corresponding 95% confidence intervals. Hypothesis tests for regression coefficients (Wald tests) were performed and expressed with p values at the significance level α = 0.05. PASW statistical software was used for all analyses (IBM Corporation, Armonk, NY, USA, version 18.0).

Knowledge and stigma were measured using scoring systems. For stigma, the responses to the corresponding questions were marked on a five-point scale (1, strongly agree; 2, agree; 3, no opinion; 4, disagree; and 5, strongly disagree), with a low score corresponding to a high degree of stigma (except for the question "Do you feel you can talk to others about your TB" where the score was first reversed before addition to its domain). The mean percentage score for stigma was calculated as 100 times the sum of scores obtained divided by the maximum scores that could be obtained. This resulted in a Cronbach α value of 0.655 in cases for which all 10 stigma questions were answered (unmarried/single patients) and 0.621 in cases for which the last stigma question was unanswered. Knowledge was computed in a less straightforward way to reflect the different degrees of difficulty for the individual questions. For questions about knowledge, contagiosity and curability of TB, the wrong answer resulted in a score of 0 and the right answer in a score of 1, except for the question of whether TB is contagious, which received a score of 2. The right answer to the question about the means of transmission resulted in a score of 3, and all other answers resulted in a score of 1. For the question about common symptoms of pulmonary TB, the score was defined as the number of marked symptoms divided by six (the total number of displayed symptoms). This gave a Cronbach α value of 0.545 for the five knowledge-related items.

## Results

### Baseline patient characteristics

We assessed 600 newly diagnosed pulmonary TB cases that were confirmed bacteriologically. Fifty of these showed TB recurrence, and 12 patients refused to participate due to poor clinical conditions. The final sample comprised 538 patients, 243 from Karakalpakstan, 179 from Tashkent, and 116 representing all 12 provinces of Uzbekistan. The characteristics of these patients are summarised in Table 1. The mean age was 40 years and there was no significant gender difference in notified TB cases. The number of cases was, however, slightly higher in men than in women. The prevalence rate was highest in men aged 25-35 years. Of the 538 patients, 231 (42.9%) practiced self-medication or consulted pharmacists with the onset of respiratory symptoms and 124 patients (54.0%) self-treated with antibiotics. The proportion of MDR-TB among new TB cases was 41% and occurred predominantly in Karakalpakstan. No cases of XDR-TB were observed among our patients.

**Table 1:** **Characteristics of pulmonary tuberculosis patients in Uzbekistan, 2014 (n = 538)**

Patient characteristics	MDR-TB (n = 243)		NON-MDR-TB (n = 295)	
	N	(%)	N	(%)
Gender				
Male	124	51.0	174	59.0
Female	119	49.0	121	41.0
M/F ratio	1.04		1.44	
Age (years)				
≤15-35	120	49.4	106	35.9
> 35	123	50.6	189	64.1
Education				
Illiterate	13	5.3	9	3.1
Primary-Secondary	206	84.8	237	80.3
University/higher	24	9.9	49	16.6
Occupation				
Employed	50	20.6	80	27.1
Healthcare worker	5	2.1	3	1.0
Unemployed	124	51.0	122	41.4
Student	7	2.9	9	3.1
Housewife	10	4.1	42	14.2
Retired	47	19.3	39	13.2
Labour migrant	27	11.1	27	9.2
Residence				
Urban	132	54.3	225	76.3
Suburban	12	5.0	41	13.9
Rural	99	40.7	29	9.8
Smoking				
Never	171	70.4	145	49.2
Current	-	-	24	8.1
Ex-smoking	72	29.6	126	42.7
Alcohol use				
Never	163	67.1	165	55.9
Past history	69	28.4	84	28.5
Moderate/Excessive	11	4.5	46	15.6
Injection drug use				
Yes	1	0.4	20	6.8
No	242	99.6	275	93.2
Comorbidities				
Diabetes mellitus	12	4.9	40	13.6
HIV positive	-	-	32	10.8
COPD/emphysema/bronchitis	42	17.3	27	9.1
Symptoms				
Cough	226	93.0	253	85.8
Fever	123	50.6	131	44.4
Loss of weight	191	78.6	191	64.7
Sputum smear-for acid-fast bacilli				
Positive	117	48.1	166	56.3
Negative	126	51.9	129	43.7
Health facility first consulted				
Pharmacy	131	53.9	100	33.9
Private clinic	4	1.6	23	7.8
Primary health center/polyclinic	42	17.3	91	30.8
TB facility	52	21.4	48	16.3
Traditional remedy	2	0.8	2	0.7
Ambulance	7	2.9	9	3.1
Other (AIDS Centre, Public hospital)	5	2.1	22	7.4
Treatment before TB diagnosis				
Self-medicated	131	53.9	100	33.9
- Self-medicated with antibiotics	90	69.0	34	34.0
Prescribed antibiotics	55	22.6	114	38.6
Time taken to reach a TB health facility				
<1/2 hour	108	44.4	46	15.6
1/2-1 hour	58	23.9	111	37.6
≥ 1 hour	77	31.7	138	46.8

TB = tuberculosis, MDR-TB = multidrug-resistant tuberculosis, HIV = human immunodeficiency virus, COPD = chronic obstructive pulmonary disease, AIDS = acquired immune deficiency syndrome.

### Patient delay

The median patient delay for all patients included in the study was 27 days [interquartile range (IQR), 6-62 days]. Delay was significantly longer in patients who abused alcohol, were HIV positive, and had specific TB symptoms of persistent cough and loss of weight (Table 2). Additionally, patients with positive sputum smear results and those who self-medicated, ordinarily with antibiotics, had longer diagnostic delay. The first healthcare provider consulted (pharmacies, private or district outpatient clinics, ambulance services) and time to reach the nearest health care facility or facility providing TB treatment were significantly associated with longer delay.

**Table 2:** **Risk factors for three delay stages of pulmonary tuberculosis patients in Uzbekistan, 2014 (n = 538)**

Predictors	N	Patient delay (median 27 days)		Health system delay (median 7 days)		Total delay (median 50 days)	
		Median	P value	Median	P value	Median	P value
Gender							
Male	298	30	0.08	6.5	0.56	49	0.8
Female	240	23.5	1	8	1	50.5	1
Age (years)							
≤ 15-35	226	25	0.3	5	0.01*	48	0.1
> 35	312	28	0.4	9	0.1	50	0.4
Education							
Illiterate	22	31	1	3	1	38.5	1
Primary-Secondary	443	25	0.5	7	0.2	50	0.6
University/higher	73	30	0.9	9	0.1	49	0.7
Occupation							
Employed	130	21	1	10	1	57	1
Healthcare worker	8	23	0.4	4	0.01*	27.5	0.8
Unemployed	246	30	0.2	5	0.001*	48	0.3
Student	16	17	0.2	14.5	0.9	36.5	0.4
Housewife	52	25	0.8	9	0.3	50	0.8
Retired	86	28	0.3	9	0.2	54	0.6
Labour migrant	54	34	0.9	7	0.02*	60.5	0.5
Residence							
Urban	357	25	1	7	1	49	1
Suburban	53	25	0.8	18	0.01*	56	0.2
Rural	128	30	0.1	4	0.1	50.5	0.9
Smoking							
Never	316	25	1	6	1	50	1
Current	24	42	0.2	8	0.2	48.5	0.9
Ex-smoking	198	29	0.3	8	0.2	49.5	0.7
Alcohol use							
Never	328	24	1	6	1	47	1
Past history	153	34	0.03*	7	0.1	54	0.1
Moderate/Excessive	57	21	0.03*	10	0.1	50	0.1
Injection drug use							
Yes	21	24	0.5	11	0.3	48	0.9
No	517	27	1	7	1	50	1
Comorbidities							
Diabetes mellitus	52	23	0.9	10	0.6	51.5	0.9
HIV positive	32	14.5	0.03*	19.5	0.02*	46.5	0.6
COPD	69	31	0.5	4	0.9	46	0.2
Symptoms							
Cough	479	29	0.008*	7	0.4	53	0.0001
Fever	254	29	0.3	7	0.8	53	0.2
Loss of weight	382	35	0.0001*	7	0.7	57	0.0001
Sputum smear-for acid-fast bacilli							
Positive	283	31	0.008*	8	0.3	54	0.006*
Negative	255	21	1	6	1	44	1
Health facility first consulted							
Pharmacy	231	41	0.0001*	6	0.0001*	56	0.0001*
Private clinic	27	14	0.02*	36	0.004*	88	0.01*
Primary health center/polyclinic	133	12	0.0001*	16	0.0001*	41	0.004*
TB facility	100	25	1	3	1	33.5	1
Traditional remedy	4	14	0.1	5	0.4	28	0.03
Ambulance	16	16	0.006*	5.5	0.4	25.5	0.05
Other (AIDS Centre, Public hospital)	27	30	0.6	6	0.4	45	0.2
Treatment before TB diagnosis							
Self-medicated	231	41	1	6	1	56	1
Self-medicated with antibiotics	124	54.5	0.0001*	4	0.03*	62.5	0.0001*
Prescribed antibiotics	169	15	0.09	25	0.0001*	58	0.02*
Time taken to reach a TB health facility							
<1/2 hour	154	24	0.5	6	0.2	39	0.3
1/2-1 hour	169	26	0.4	8	0.4	55	0.4
≥ 1 hour	215	29	0.04*	7	0.6	51	0.04*

HIV = human immunodeficiency virus, COPD = chronic obstructive pulmonary disease, AIDS = acquired immune deficiency syndrome. *p <0.05.

### Health system delay

The median health system delay of 7 days (IQR, 1-32 days) seemed to be insignificant (Table 2). The health system delay was associated with younger age, unemployment, or labour migration, for those who had recently worked abroad (p = 0.001 and p = 0.02, respectively). Being a health care worker was a strong risk factor (p = 0.01) for health system delay, but could not be fully assessed since there were only eight (1.5%) healthcare workers and all of them had delayed diagnosis. Furthermore, patients who had longer health system delay also were HIV-positive, stigmatised by TB disease, and prescribed antimicrobials.

### Total delay

The median total delay was nearly 2 months (50 days, IQR, 22-92 days), and 228 of 538 patients (42%) experienced a delay of ≥3 months. Patients with a cough, loss of weight, who self-medicated, were prescribed anti-TB medications by non-TB physicians, from distant location to healthcare facility, or facility providing TB treatment experienced longer delays in the diagnosis and treatment of TB.

Further multivariate analysis revealed that self-medication and initial consultation with a private facility predicted long patient delay, while administration of antimicrobial therapy as well as seeking care at private clinics were risk factors for health system delay (Table 3). Cough, self-medication, and private and primary healthcare facilities were associated with long total delay.

**Table 3:** **Multiple logistic regression analysis of risk factors for three delay stages of pulmonary tuberculosis patients in Uzbekistan, 2014 (n = 538)**

Predictors	Patient delay; OR (95%CI)	Health system delay; OR (95%CI)	Total delay; OR (95%CI)
Cough	0.72 (0.38 to 1.39)	1.02 (0.53 to 1.96)	4.67 (1.23 to 17.1)*
Loss of weight	0.25 (0.16 to 0.4)*	1.04 (0.65 to 1.67)	0.42 (0.27 to 0.65)*
Sputum positive	0.60 (0.40 to 0.89)*	1.12 (0.73 to 1.69)	0.77 (0.52 to 1.13)
Self-medication	1.53 (1.01 to 2.33)*	0.91 (0.53 to 1.53)	1.58 (1.12 to 2.23)*
Self-medication with antibiotics	0.73 (0.40 to 1.31)	1.31 (0.69 to 2.45)	0.94 (0.53 to 1.68)
Prescribed antibiotics	N/A	2.19 (1.18 to 4.09)*	0.5 (0.31 to 0.81)
Primary health center/ Polyclinic	1.68 (0.49 to 5.63)	3.09 (0.61 to 15.74)	5.53 (1.29 to 23.72)*
Private clinic	2.96 (1.49 to 5.88)*	2.87 (1.83 to 4.54)*	4.67 (1.27 to 17.11)*

OR = odds ratio, CI = confidence interval, N/A = not applicable.

*p <0.05.

Untreated MDR-TB is highly contagious; therefore, we defined the extent of delay and evaluated risk factors among patients with and without MDR-TB. Among MDR-TB patients, there was a slightly longer median patient delay of 36 days, healthcare delay was 8 days, and total delay was 47 days. There were no differences between the two groups for predictors of diagnostic delay. However, high social stigma prevailed in patients with MDR-TB (odds ratio 1.39, 95% confidence interval 0.74-2.62).

## Discussion

We found that the median total delay time from onset of symptoms until diagnosis and initiation of anti-TB treatment was 50 days and was mainly contributed to by patient delay of 27 days. The healthcare system delay of 7 days was significantly shorter than patient delay. These data are similar to the results of recent studies from Georgia and Ukraine [14],[16].

Importantly, in our study, 79 of 228 cases with >2 months total delay had MDR-TB, for which early and accurate diagnosis is critical to timely initiation of effective treatment.

The main factors associated with total delay in a cohort of TB patients after adjusted multivariate analysis were self-medication, seeking initial care from a primary health facility or the private sector, and cough.

Almost half of the patients used unprescribed antimicrobials before diagnosis and treatment of TB. It is known that self-medication with antibiotics can delay and mask the correct diagnosis of infectious disease [17]. Several studies have reported that antibiotic exposure, in particular fluoroquinolones, can delay the diagnosis of TB and initiation of anti-TB treatment [18]-[20]. However, in our study, the contribution of the class of antibiotics was not investigated.

In addition, both first- and second-line anti-TB drugs are available without prescription, which encourages self-treatment and the uncontrolled use of these drugs may contribute to higher levels of resistance, with a consequent deleterious impact on treatment success [21],[22]. Self-treatment only temporarily relieved the symptoms and resulted in exhaustion, weakness and the need for transportation by ambulance.

Importantly, care seeking from a primary health centre/polyclinic was associated with a delay in diagnosis that might have been due to failure to comply with recommended diagnostic standards. Patients managed in primary health centres were repeatedly treated with antibiotics (including fluoroquinolones) for upper respiratory infection or presumed community-acquired pneumonia. Prolonged delay in diagnosis ranging from 1 to 12 months was seen in 8% of patients first approaching public polyclinics. Inadequate case detection at primary health care contributes to low treatment success rate and an extended period of disease transmission, particularly MDR-TB. Of particular concern were private physicians who were most often consulted first by patients living in distant places. As in some studies from developing countries [23],[24], private physicians tend to deviate from recommended TB management guidelines and rely on chest radiography rather than referral of patients for sputum microscopy or monitoring treatment [25].

HIV infection was significantly associated with longer patient and health system delay. This was possibly because symptoms were less specific and might have been considered by the patients to be associated with HIV. Despite all HIV-infected patients being routinely screened for latent and active TB infection, the interpretation of test results might be complicated by a higher incidence of false-positive results (e.g., negative tuberculin skin test, negative sputum and normal chest x-ray findings), and lack of specificity of symptoms [26].

The reason that cough was associated with delayed TB diagnosis is not clear. It might be that patients considered it as a transient symptom from an upper respiratory illness, hence initiating self-treatment lasting until deterioration and manifestation of other specific symptoms. Furthermore, timely referral to healthcare facilities was challenging for work migrants due to limited access to health care, financial constraints, poor health literacy, and fear of deportation after positive TB diagnosis.

Although awareness and knowledge of TB were high in Karakalpakstan, the extent of total delay was similar across regions, yet patient delay was longer in Karakalpakstan, possibly due to greater stigmatisation, fear and belief in the incurability of the disease. Approximately 30% of those with diagnostic delay in Karakalpakstan had relatives and friends with MDR-TB or even experienced the death of a family member, and the feeling of hopelessness was overpowering. This is in line with the findings of Kuznetsov et al. [27], who described hopelessness as a basis for TB diagnostic delay in the Arkhangelsk region.

In agreement with previous studies on delay in TB diagnosis [28],[29], we found that current smoking was associated with longer patient delay. This could be explained by the fact that some TB symptoms can be confused with other smoking-related conditions [30]. For example, chronic obstructive disease was found to be a comorbid condition in 14% of smokers diagnosed with TB and could mask the symptoms of TB, resulting in delayed TB diagnosis. Alcohol abuse was another risk factor for delayed TB diagnosis.

There were several limitations to our study. First, recall bias may have influenced our results, because the onset of first symptoms may have been inaccurately reported by the patients. In order to minimise patient recall bias, we encouraged physicians to check patient medical cards when completing the questionnaire. Second, a lack of information on the class of antibiotic used for self-medication, as well as that prescribed by the physician, meant that fluoroquinolone use may have been associated with delay in diagnosis of TB.

Although we investigated the risk factors associated with delayed diagnosis and treatment of TB, there are clinical consequences of late TB diagnosis. Further studies are required to assess morbidity, mortality, treatment success, outcome of previous antibiotic treatment, risk of transmission, and development of active TB associated with delay in initiation of treatment.

However, the significance of delay for treatment success remains unclear.

## Conclusion

TB diagnostic and treatment delay should be reduced to the least possible time interval. There is a need to decrease TB stigma and promote public awareness of TB curability and the importance of early referral to health services. An essential step is to improve the diagnostic awareness among private and primary care practitioners. A high index of suspicion of TB should be maintained in public and private practitioners and an appropriate diagnostic work-up should be performed. Regulations prohibiting the dispensing of antibiotics, including anti-TB medicines, without prescription should be enforced to prevent further development of drug- resistance.

## Authors' contributions

TB was a principle investigator responsible for design and conception of the study, data collection, and interpretation of data, and wrote the manuscript. DK, MT, ZT and MK participated in study design, coordination, and data collection and drafted the manuscript. JDT conducted statistical analysis and drafted the manuscript. JV participated in study design and coordination, and review of the manuscript. All the authors read and approved the final manuscript.
